# Supplementary material for: The interactome of intact mitochondria by cross-linking mass spectrometry provides evidence for coexisting respiratory supercomplexes
Source: Mol Cell Proteomics. 2017 Dec 8;17(2):216–32. doi: 10.1074/mcp.RA117.000470 (PMC5795388; doi:10.1074/mcp.RA117.000470)
Supplement: Supplemental Data [file supp_RA117.000470_133922_0_supp_23978_fzffwf.zip › spectra_annotation/mito_spectra_annotation/244-1-9-1-7-1.pdf]

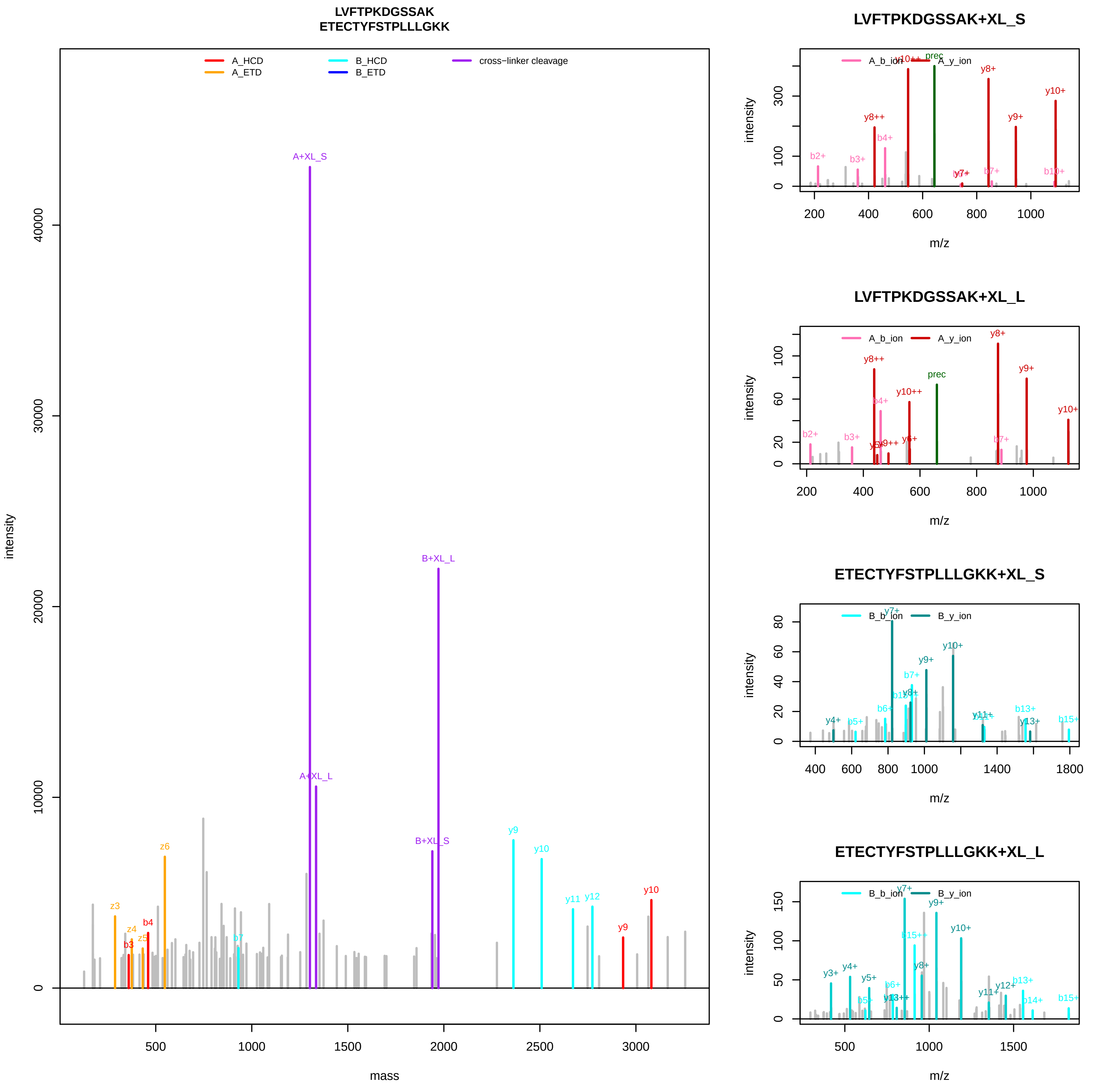
LVFTPKDGSSAK
ETECTYFSTPLLLGKK
A_HCD
A_ETD
B_HCD
B_ETD
cross−linker cleavage
intensity
mass
0
10000
20000
30000
40000
500
1000
1500
2000
2500
3000
A+XL_S
B+XL_L
A+XL_L
B+XL_S
z3
z4
z5
z6
b3
b4
b7
y9
y10
y11
y12
y9
y10
LVFTPKDGSSAK+XL_S
A_b_ion
A_y_ion
prec
b2+
b3+
b4+
b7+
b10+
y7+
y8++
y8+
y9+
y10+
y10++
m/z
LVFTPKDGSSAK+XL_L
A_b_ion
A_y_ion
b2+
b3+
b4+
b7+
y5+
y8++
y8+
y9++
y9+
y6+
y10++
y10+
prec
m/z
ETECTYFSTPLLLGKK+XL_S
B_b_ion
B_y_ion
y4+
b5+
b6+
b7+
y7+
y8+
y9+
y10+
y11+
b13+
y13+
b15+
m/z
ETECTYFSTPLLLGKK+XL_L
B_b_ion
B_y_ion
y3+
y4+
y5+
b5+
b6+
y7+
b15++
y8+
y9+
y10+
y11+
y12+
b13+
b14+
b15+
y13++
m/z
